# Effect of Foliar Micronutrients (B, Mn, Fe, Zn) on Maize Grain Yield, Micronutrient Recovery, Uptake, and Partitioning

**DOI:** 10.3390/plants10030528

**Published:** 2021-03-11

**Authors:** Zachary P. Stewart, Ellen T. Paparozzi, Charles S. Wortmann, Prakash Kumar Jha, Charles A. Shapiro

**Affiliations:** 1 Center for Agriculture-Led Growth, Bureau for Resilience and Food Security, United States Agency for International Development, 1300 Pennsylvania Ave NW, Washington, DC 20004, USA; 2 Department of Agronomy and Horticulture, University of Nebraska, Lincoln, NE 68583-0915, USA; etp1@unl.edu (E.T.P.); cwortmann2@unl.edu (C.S.W.); cshapiro@unl.edu (C.A.S.); 3 Feed the Future Innovation Lab for Collaborative Research on Sustainable Intensification, Kansas State University, Manhattan, KS 66506-4004, USA; pjha@ksu.edu; 4 Department of Agronomy, Kansas State University, Manhattan, KS 66506, USA; 5 Haskell Agriculture Laboratory, 57905 866 Road, Concord, NE 68728-2828, USA

**Keywords:** foliar, maize, corn, boron, iron, manganese, zinc, uptake, partitioning, translocation

## Abstract

Timing of micronutrient demand and acquisition by maize (*Zea mays* L.) is nutrient specific and associated with key vegetative and reproductive growth stages. The objective of this study was to determine the fate of foliar-applied B, Fe, Mn, Zn, and Fe/Zn together, evaluate the effect of foliar micronutrients applied at multiple rates and growth stages on maize grain yield, and determine their apparent nutrient recovery efficiency (ANR). Five Randomized Complete Block Design (RCBD) experiments were conducted in 2014 and 2015 at five locations across Nebraska. Total dry matter was collected at 5–6 stages, and separated into leaves, stalk, and reproductive tissue as appropriate to determine micronutrient uptake, partitioning, and translocation. Foliar B, Mn, Zn, and Fe/Zn had no effect on grain yield for most application time by rate levels, though, at the foliar Mn site, there was a 19% yield increase due to a V18 application of 0.73 kg Mn ha^−1^ which corresponded with reduced Mn uptake in maize grown in control plots. At the foliar Zn site, there was 4.5% decrease in yield due to a split foliar application of 0.84 kg Zn ha^−1^ total, applied at V11 and V15 stage, which increased leaf Zn concentrations greater than the established toxic level. Only the Fe site had consistent grain yield response and was the only experiment that had visual signs of micronutrient deficiency. Regardless of application time from V6 to R2, there was a 13.5–14.6% increase in grain yield due to 0.22 kg Fe ha^−1^ foliar application. Most micronutrients had limited or no translocation, however, early season applications of B, prior to V10, had significant mobilization to reproductive tissues at or after VT. Foliar Mn, Zn, and B application had ANR LSmeans of 9.5, 16.9, and 2.5%, respectively, whereas the Fe/Zn mix had negative ANR LSmeans of −9.1% Fe and −1.3% Zn which indicate suppression. These data highlight the importance of confirming a micronutrient deficiency prior to foliar application, guide specific growth stages to target with specific micronutrients, track the fate of foliar-applied micronutrients, and describe the variable effect of foliar-applied micronutrients on grain yield.

## 1. Introduction

Best nutrient management practices require synchronous nutrient application with plant demand and nutrient uptake. Nutrient demand and acquisition in maize (*Zea Mays* L.) is associated with key vegetative or reproductive growth stages [1]. To maximize fertilizer uptake and utilization, it is essential to apply or have nutrients available at the time of greatest demand [2]. Bender et al. [1] highlighted the need to develop recommendations of timebound nutrient applications to sync up nutrient’s uptake and mobilization during periods of high plant uptake for modern maize hybrids. This is especially critical for micronutrient applications, as micronutrients are needed in relatively small but critical amounts by maize at specific growth stages during the growing season [3]. For most nutrients, seasonal uptake in maize is sigmoidal with the maximum rates of nutrient uptake occurring between V10 and V14 and plateauing at VT/R1. As much as two-thirds of boron (B), manganese (Mn), and iron (Fe) uptake occurs before reproductive growth stages compared to only one-half of zinc (Zn) uptake. For Zn, more than 70% of Zn uptake occurs slightly later during one-third of the growing season in late vegetative and early reproductive growth. B uptake follows a similar trend with 65% of B uptake occurring during one-fifth of the growing season during late vegetative growth. Fe uptake has two periods of critical accumulation: between V10 and V14, and after R4, whereas Mn uptake is more gradual with a majority of Mn uptake occurring from V10 to R4 [1]. Each of these periods of high micronutrient uptake and demand should be targeted for micronutrient specific application.

The application of foliar micronutrients to avoid micronutrient deficiencies for deficient soils is a common practice worldwide [4,5]. Foliar applications have several benefits which often make this method an ideal choice for application of micronutrients over soil applications. These benefits include: (1) the avoidance of interaction with soil properties; (2) in-season application during high plant demand; (3) rapid plant response to application; and (4) cost effective for one time application [6]. However, despite high soil fertility in Nebraska, maize has a high rate of nutrient uptake during specific growth stages and demand may exceed supply [1]. Most research on foliar-applied B, Mn, Fe, and Zn on maize have focused on single application times, both in deficient and sufficient field locations and have reported inconsistent and mixed results [7,8,9,10,11,12,13,14,15,16]. Potarzycki and Grzebisz [14] reported an increase in maize grain yield of nearly 18% for a three-year average with the application of 1.0 to 1.5 kg foliar Zn ha^−1^ and Nelson and Meinhardt [13] reported an increase in maize grain yield of 6% for a three-year average with the application of 0.56 kg foliar B ha^−1^ while many others show no significant yield increase. Differential yield responses for each of these studies were not associated with a consistent predictor such as low soil or plant nutrient concentration, soil organic matter, pH, or texture.

There is a need to determine when to apply a foliar application of micronutrients to maximize each nutrient’s uptake and mobilization characteristics. Knowledge of the dynamics of micronutrient accumulation to sink organs and the fate of foliar-applied micronutrients at specific growth stages would provide a useful tool to deliver micronutrients more efficiently to meet demand. For many crops, soil micronutrient recovery efficiency ranges from only 5–10%, however there is a lack of data on the recovery efficiency of foliar-applied micronutrients applied at different rates and growth stages in maize production [17,18,19]. As leaves develop, they transition from nutrient importing sink organs to nutrient exporting source organs. Mature leaves also become less capable of importing nutrients while immature leaves are entirely dependent on the import of nutrients and are physiologically incapable of exporting nutrients [20]. It can be theorized that applications to immature leaves would be more likely to take-up the applied nutrients but less likely to be a source of the micronutrients to other plant organs, at least until maturity. Applications to older mature leaves may have reduced recovery efficiency but may be more capable of becoming a source of foliar absorbed nutrients. These data would be valuable for understanding variation in yield response to specific nutrients applied at specific times and further direct application guidelines.

Studies have shown that cuticular penetration of foliar-applied nutrients is largely a diffusion process, though ions can also be transported into the leaf by facilitated diffusion [21,22]. Foliar solutes can also enter the leaf through cuticle cracks and imperfections, the stomata, leaf hairs, trichomes, and other specialized epidermal cells [6]. After passing through the cuticle, nutrients can accumulate in the intercellular space, a region outside of the cell wall of the leaf before moving to metabolically active sink cells [23]. Once inside the leaf, nutrients have two pathways to reach vascular tissues: apoplastic or symplastic transport. The free space between cells provides a pathway for apoplastic movement of nutrients. Cells can also actively or passively transport nutrients through the cytoplasmic continuum, (specific ion channels and aquaporins) thereby directly moving nutrients from cell to cell through symplastic transport [23]. The rate of translocation depends on the specific nutrient and the plant species [24]. Once assimilated into a metabolic role, micronutrients have limited remobilization to other new growth plant organs which makes timing of micronutrient supply with demand even more important [6]. There is also a lack of understanding regarding the fate of micronutrients applied to the leaf surface of maize. Moreover, the associated puzzle remains unresolved. Do the applied micronutrients stay in the leaf only having localized effect or do they mobilize to other metabolically active sink cells in other plant tissues? Do micronutrients applied to older, more mature leaves have similar effects as micronutrients applied earlier in the growing season to immature leaves? To get answers for these questions, synergistic experiments with time bound application of micronutrients is needed.

B, Fe, Mn, and Zn application were considered most agronomically important to Nebraska maize production based on a soil and plant tissue sampling survey [16] and agronomic testing laboratory data and thus were evaluated. The objective of this study was to determine the fate of micronutrients applied to the leaf surface, the recovery efficiency of the foliar-applied micronutrients, and evaluate the effect of foliar-applied micronutrients on maize grain yield when applied at key growth stages at high yielding locations. Specifically, in the case of maize plants being low (i.e., near critical levels but not necessarily below) in plant tissue concentrations without any confirmed micronutrient deficiency. These data will be useful to compare to the conventional deficiency correction theory to the temporal deficiency theory.

## 2. Materials and Methods

### 2.1. Experimental Design and Site Selection

**Figure 1:**
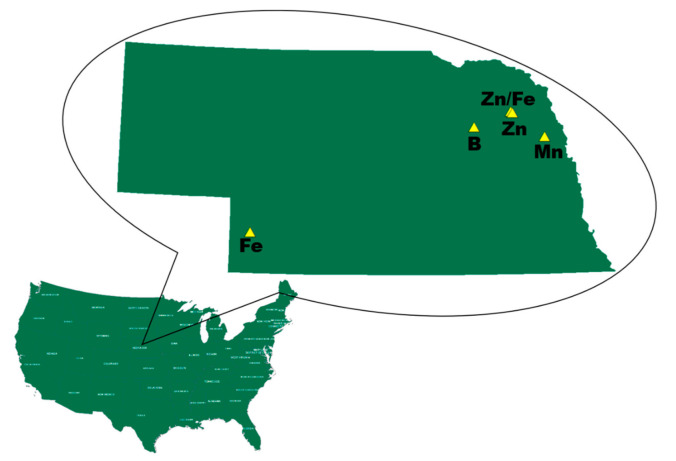
Locations of field experiments

Five multi-location randomized complete block design (RCBD) field trials were performed in 2014 and 2015 in Nebraska (Table 1, Figure 1). Replications at each location were blocked by soil type using Web Soil Survey (USDA, 2013). The maize vegetative stages for foliar application are explained by Abendroth et al. (Figure S1) [25]. Locations were selected prior to foliar treatment based on having a past maize yield history more than 12.5 Mg ha^−1^ and having spring soil and/or plant tissue samples (V5–V6) [24] indicating “deficient” or “low” levels of the target micronutrient according to industry standards and Mills et al. [26] (Table 2). All locations had nine treatment combinations of different treatment rates and application times (Table 3) and nine replications for yield and six replications for whole plant sampling except the foliar Fe/Zn location, which had twelve treatment combinations and four replications for yield and whole plant sampling. The upper most fully collared leaf from nine plants (V5–V6) were combined for each plant tissue sample in each block. Similarly, nine soil cores, 20 cm deep, were collected from each block and combined for each soil sample in each block (Oakfield Apparatus Company, Oakfield, WI, USA, 2.5 cm diameter).

Pre-season nitrogen applications varied by source and rate, but all locations had applied nitrogen at a rate sufficient for 13–16.0 Mg ha^−1^ maize grain production [27]. Additionally, a one-time application of 100 kg N ha^−1^ was applied to the Mn, Zn only, and Fe/Zn locations in the form of urea at R1 in response to mid-season heavy rainfall and hail damage at these locations. This late season application of N was supplied to remediate any N losses due to adverse weather conditions. Three of the five locations were fully irrigated by center pivot irrigation and two locations had no irrigation; however, the two locations without irrigation had rainfall approximately 250 mm greater than their ten-year averages. All locations had rainfall greater than their ten-year averages, and maize as their previous crop with 0.76 m row spacing (Table 1). Other agronomic practices were chosen by the producer as to best mediate pest, weed, or other fertility issues. Management practices and relevant site information can be found in Table 1 and Table 2.

### 2.2. Micronutrient Foliar Treatments

Micronutrient foliar treatments were assigned at each site based on micronutrient recommendations from in-season V5–6 plant tissue samples and/or spring soil samples (0–20 cm) (Table 2 and Table 3). Foliar treatments were applied by backpack sprayer (R and D Sprayers, Opelousas, LA, USA) using a XR 11003VS flat-fan nozzle tip (TeeJet Technologies, Spray Systems Co., Wheaton, IL, USA) at 140 L ha^−1^ and a pressure of 207 kPa. Foliar treatments were applied to four row plots 9.1 m long by 3.05 m wide (i.e., four rows with 0.76 m spacing) approximately 0.3 m above the canopy. The center two rows were harvested for grain yield determination, and destructive whole plant samples were collected from the outer two rows. A buffer row and 0.6 m alleys bordered each plot to prevent cross-contamination by spray drift. Micronutrient foliar treatments were applied at three growth stages (1: early (V6–V11), 2: middle (V15–V18), 3: late (R1–R2)) and two rates (1X rate: lower level of industry recommendation and 2X rate: upper level of industry recommendation) (Table 3). There was always at least one day between foliar nutrient application and either a rainfall or irrigation event.

These rates were within the “usual application rates range” on a nutrient bases for B and Mn (i.e., <1 kg ha^−1^ B and 1–10 kg ha^−1^ Mn) and slightly below for Zn and Fe (i.e., 1–10 kg ha^−1^ Zn or Fe) as reported by Mortvedt [18]. The Fe/Zn location had treatments applied at four growth stages which added a R4 application. The foliar micronutrient treatments were: MAX-IN^®^ Boron (WinField Solutions: St. Paul, MN, USA) 8.0% B derived from boric acid, MAX-IN^®^ Ultra Manganese (WinField Solutions: St. Paul, MN, USA) 15.62% MnSO4, Origin^®^ Zinc 9% (WinField Solutions: St. Paul, MN, USA) 9.0% ZnEDTA (zinc-ethylenediaminetriacetate), and ULTRA-CHE IRON 4.5% HEDTA (WinField Solutions: St. Paul, MN, USA) 4.5% FeHEDTA (iron-hydroxyethylenediaminetriacetate). All treatments contained CornSorb^®^ proprietary surfactants, saccharides, and antifoaming solvents. The Fe/Zn location used a custom blend of both ULTRA-CHE IRON 4.5% HEDTA (4.5% FeHEDTA) and Origin^®^ Zinc 9% (9.0% ZnEDTA). The micronutrient foliar treatment rates, mass of applied nutrient, and concentrations are provided in Table S1. Control plots received the same management practices in all regards except did not receive a foliar treatment.

### 2.3. Whole Plant Sampling

To evaluate the fate, partitioning, and mobility of the micronutrient foliar treatments, six plants were sampled from each plot at five growth stages: (1) V6–V7 prior to foliar treatments, (2) V13–V15 following the “early (T1)” foliar treatments, (3) V17-VT following the “middle (T2)” foliar treatments. (4) R2–R3 following the “late (T3)” foliar treatments, (5) R6 final collection (following a R4 application (T4) only at the Fe/Zn location). Six plants were cut at the soil surface from 8:00 to 11:00 AM and separated into four components and are reported as stalk, leaf, reproductive (tassel, cob, and husk), and grain tissues [1]. Each component was weighed no longer than five hours following harvest. Stalk tissue for reproductive stage plants were shredded with a commercial chipper (MacKissic Inc., Mighty Mac 12P Shredder-Chipper) to obtain a representative sub-sample, which reduced the amount of matter needed for drying, and insured uniform dry-down. Leaf, reproductive portion, and grain from six plant samples were not sub-sampled to keep the entire dry matter uniform dry-down. Partitioned samples were oven-dried at 65 °C to constant mass, weighed, and foliage analyzed for nutrient concentrations (Midwest Laboratories, Omaha, NE). A ratio of water content to dry matter content was calculated using the initial subsample weight and the final dry sub-sample weight. This ratio was used to calculate the weight of dry matter in the initial harvested stalk sample.

All units are expressed on a dry weight (0 g kg^−1^ water content) basis. Grain nutrient analysis was performed from the six partitioned plants, whereas yield estimates were harvested at physiological maturity (R6) from the middle two rows of each plot with a plot combine (Almaco, SPC40) and standardized to 155 g kg^−1^ water content.

### 2.4. Laboratory Analysis

Laboratory analysis of plant tissue phosphorous (P), potassium (K), sulfur (S), calcium (Ca), Fe, Mn, Zn, and B were completed using microwave nitric acid digestion and concentrations were determined using inductively coupled plasma (ICP) spectroscopy. The percent nitrogen (N) was determined using the Dumas Method with a Leco FP-428 (Horwitz and Latimer Jr, 1920). Laboratory analysis of grain tissue B, Ca, Cu, Fe, Mg, Mn, P, K, Na, S, and Zn was prepared using the ME PROC 69 methodology. The analysis of these data followed ME PROC 29 methodology [28]. Samples were treated with a combination of heat and mineral acids to dissolve the minerals and destroy organic materials. The extract was analyzed for mineral content by Inductively Coupled Argon Plasma Emission Spectrometer (ICAP-ES). Boron was not detected in grain samples at the 3 mg kg^−1^ detection limit. Spring soil samples (0–20 cm) were analyzed for Mn, Fe, and Zn concentration using DPTA (diethylenetriaminepentaacetic acid) extraction along with ICAP-ES detection. B concentration was measured using DPTA and used sorbitol ICAP-ES detection.

Apparent nutrient recovery (ANR) at the end of the growing season (R6) in the whole plant, above the soil surface, was calculated to reflect the efficiency of maize to recover the applied foliar micronutrient(s).

ANR (%) = [(nutrient uptake fertilized (g ha^−1^) − nutrient uptake control (g ha^−1^))/(quantity of nutrient applied (g ha^−1^))] × 100.

### 2.5. Statistical Analysis

Grain yield, biomass, nutrient uptake, and nutrient concentration for all partitioned tissues (i.e., leaf, stalk, reproductive, and grain), and ANR were analyzed using PROC GLIMMIX SAS 9.3 software (SAS Institute Inc., 100 SAS Campus Drive, Cary, NC, USA) [29]. Block was designated as a random effect. A mean comparison test using the Dunnett Adjustment was used to compare treatment effects to the “control.” Orthogonal contrasts for grain yield and nutrient quantity in partitioned and total foliage at differing growth stages and at individual locations were performed using the CONTRAST statement of SAS and were planned and selected prior to analysis. Nutrient uptake and partitioning graphs were generated by SigmaPlot (SigmaPlot v11.0; Systat Software Inc. San Jose, CA, USA). Means generated from Excel (Microsoft Excel 2013, Microsoft Corp. Santa Rosa, CA) were imported into SigmaPlot and uptake curves were generated with the simple spline curve option with smoothed data points like Bender et al. [1]. An ANOVA for overall treatment effects on total uptake was conducted for non-applied nutrients at each location (N, P, K, S, Mg, Ca, Mn, B, Fe, and Zn). The ANOVA test for each non-foliar-applied nutrient (i.e., N, P, K, Mg, Ca, S, Cu, and Na) at each location reported no significant treatment main effects at *p* < 0.05 hence no further analysis was conducted.

## 3. Results and Discussion

### 3.1. Trial Locations and Selection

All selected trial locations had V5–V6 plant tissue concentrations of the applied micronutrient near but not below critical levels except in the case of Fe at the combined Fe and Zn location (Winside) which was in excess (Table 2). Plant tissue macronutrient concentrations were also above critical concentrations in V5–V6 plant tissue (Table 2) as reported by Mills et al. [26]. Thus, indicating macronutrients were likely not limiting according to Liebig’s Law of the Minimum [30]. All soil nutrient concentrations were also above critical levels as reported by Wortmann [31] and Ward [32] though lime would be recommended at the foliar B, Zn, and Fe/Zn mix sites (Table 2). All locations also had a history of being relatively high yielding (i.e., maize yield history in excess of 12.5 Mg ha^−1^). Only the 2015 foliar Fe application location had visual signs of deficiency (i.e., interveinal chlorosis in the upper most new growth leaves) though neither soil or plant analysis Fe was below critical levels of 4.5 mg Fe kg^−1^ soil as reported by Ward [32] and 10 mg Fe kg^−1^ leaf tissue for maize as reported by Mills et al. [26] (i.e., 4.9 mg Fe kg^−1^ and 181 mg Fe kg^−1^, respectively). Soil pH was alkaline (i.e., 7.5) which likely contributed to reduced micronutrient availability and the subsequent visual signs of deficiency. This location was fully irrigated and high yielding (i.e., 14.2 Mg ha^−1^ in control plots) (Table 3).

The foliar B location for control plots had V6 leaf tissue below critical B concentrations (i.e., 4.0 mg B kg^−1^ in leaf tissue for maize prior to tassel) as reported by Mills et al. [26] (Table 4a) but V5 leaf tissue analysis reported B at 6.0 mg B kg^−1^ (Table 2), and there were no visual signs of B deficiency. The soil DTPA extractable B concentration of 0.7 mg B kg^−1^ was also marginally above the 0.5 mg B kg^−1^ critical level as reported by Ward [32]. This location was fully irrigated and was high yielding (i.e., 14.9 Mg ha^−1^ in control plots) (Table 4). The location receiving foliar Zn only had V5 plant tissue concentrations of 22.3 mg Zn kg^−1^, which were above the Zn critical level of 15 mg Zn kg^−1^ (i.e., as reported by Mills et al. [26]. The soil analysis for this location reported DTPA extracted Zn at 0.9 mg Zn kg^−1^, which was also marginally above the critical level of 0.75 mg Zn kg^−1^ as reported by Voss [33], Wortmann et al. [34], and Ward [32]. This location had hail on July 7th with an estimated 5–10% yield reduction [35] but was still high yielding (13.7 Mg ha^−1^ in control plots) (Table 3).

The location receiving foliar Mn application was well above the 15 mg Mn kg^−1^ leaf tissue critical value at V5 (i.e., 62.0 mg Mn kg^−1^), however, plant tissue Mn was relatively low compared to other sampled locations in Nebraska (Table 2). DTPA extracted soil Mn was 22 mg kg^−1^, which was above the 2.0 mg kg^−1^ critical soil level as reported by Ward [32]. The foliar Mn location had heavy rainfall (i.e., 202 mm from June 20th to July 4th) and standing water for nearly two weeks which correlated with a consistent drop in Mn plant tissue concentration and Mn uptake (Table 4b and Table 5b) prior to the foliar Mn treatment. Although this location had a history of yields more than 12.5 Mg ha^−1^, heavy rainfall reduced the control yield to 7.9 Mg ha^−1^ (Table 4). The location receiving both foliar Fe and Zn had plant tissue Zn at the Zn critical level of 20 mg Zn kg^−1^, however, plant tissue Fe, 403 mg Fe kg^−1^, was greater than the upper value of the Fe sufficiency range of 50–250 mg Fe kg^−1^ as reported by Mills et al. [26]. Both soil Zn and Fe (i.e., 1.4 mg Zn kg^−1^ and 110 mg Fe kg^−1^) were above the soil critical levels of 0.75 and 4.5 as reported by Ward [32]. This location also had hail on July 7th with an estimated 5–10% yield reduction [35] but was still high yielding (12.7 Mg ha^−1^ in control plots) (Table 3).

Though none of the targeted micronutrients were below critical levels during early season leaf tissue samples (V5–V6), it has been shown that these nutrients fluctuate throughout the day within the growing season due to environmental stresses and in some samples, micronutrient concentrations fell below their respective critical levels (i.e., B location) during the growing season [36]. Leaf tissue nutrient concentrations are known to fluctuate throughout the growing season due to environmental factors (i.e., soil water, temperature) between periods of adequate soil supply of micronutrients and periods of insufficient soil supply of the applied micronutrient [36]. Further, the time of day for plant sampling effects plant concentrations of Fe, Mn, and Zn and can decrease nutrient concentration by as much as 243, 26, and 5 mg kg^−1^, respectively, due to mid-day sampling as compared to morning sampling, but time of day has less effect on B concentration [36] and thus, these locations were still considered suitable sites that met the study objectives. Following the deficiency correction hypothesis, yield increases may not be expected if micronutrient concentrations do not fall below critical levels at any point during the growing season.

### 3.2. Effect of Foliar Micronutrients on Grain Yield in Relationship with Plant Nutrient Concentrations

The foliar-applied B, Mn, and Zn experiments showed these nutrients had limited effect on grain yield for most application time by rate level combinations though there was a 19% yield increase (*p* = 0.006) due to a V18 application of 0.73 kg Mn ha^−1^ and a 4.5% yield decrease (*p* = 0.02) due to a split application of foliar 0.84 kg Zn ha^−1^ applied at V11 and V15 compared to the control (Table 3). The foliar Fe location (Imperial) had an average soil pH of 7.5, low plant tissue and soil Fe concentrations, and showed visual signs of deficiency throughout the entirety of the trial growing season. Yields were consistently increased due to either a single foliar application or a split application of foliar 0.22 kg Fe ha^−1^ (i.e., 2×). There was a 14.6% increase (*p* = 0.04) due to the 2× application at V6 (i.e., T1R2), a 14.2% increase (*p* = 0.04) due to a split application of the 2× rate at V6 and V15 (i.e., T1R1 and T2R1), and a 13.5% increase (*p* = 0.05) due the 2× application at R2 (i.e., T3R2). The 2× rate consistently outperformed the 1× rate across all treatments and time of application did not have a significant effect on yield (*p* < 0.05). Further, the single 1× application of foliar Fe (i.e., 0.11 kg Fe ha^−1^) had consistently greater yield than the control and less than the 2× rate but not significantly. As there were only three Fe rates no response function could be calculated. Therefore, it is unknown if a greater rate of Fe application would have greater response. These data highlight the importance of confirming a micronutrient deficiency prior to applying a foliar micronutrient treatment and is consistent with the deficiency correction theory.

**Figure 2:**
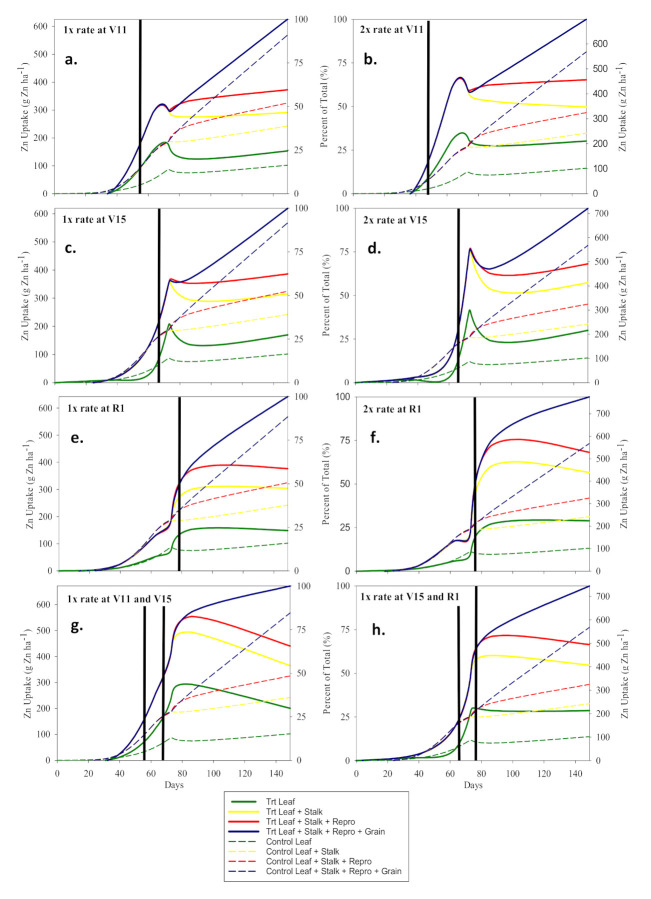
Zn uptake (g Zn ha^−1^) and partitioning graphs. Solid and dashed lines represent foliar Zn treated plots and control plot, respectively. Solid vertical lines represent the time of application expressed as days after sowing. (**a**–**h**) corresponds with the treatments.

At the B location (Meadow Grove), there were no significant yield effects. The B location for the control plots reported V6 leaf tissue below critical B concentrations (i.e., 4.0 mg B kg^−1^ in leaf tissue for maize prior to tassel) as reported by Mills et al. [26]. Additionally, the B location control plots had end of the season B leaf concentrations (R6) below critical B concentrations (i.e., 5.0 mg B kg^−1^ in leaf tissue for maize after tassel) (Table 5a). Both the T2R1 and T3R2 treatments increased B concentrations above the critical level (6.0 and 5.7 mg B kg^−1^, respectively) in leaf tissue but this was not associated with a significant increase in grain yield (*p* < 0.05). The Mn location (Oakland) had significant grain yield increase due to the T2R1 treatment (i.e., V18 application of foliar 0.73 kg Mn ha^−1^). This yield increase may be due to heavy rainfall (i.e., 202 mm rainfall from 20 June–4 July) where there was standing water on the experiment for approximately 2 weeks (Table 1) causing reduced uptake and concentration of Mn (unpublisehd data) prior to the foliar Mn treatment. Figure 2d indicates that the V18 treatments were applied during the period of reduced soil supply, therefore, preventing the dip in Mn uptake as seen in the control plots and all other non V18 treated plots. The concentration of Mn in the leaf tissue following the V18 treatment significantly increased the control from 83.5 mg Mn kg^−1^ to 108.7 mg Mn kg^−1^; however, both leaf concentrations are well within the sufficiency range for maize prior to tassel (i.e., 15–300 mg leaf Mn kg^−1^) as reported by Mills et al. [26]. Though each of the V18 treatments had yields greater than the control, none was significantly greater than the control at *p* < 0.05.

Applying the right rate at the right time during periods of insufficient soil Mn supply was likely the primary driver for the significant 19% increase of 1.52 Mg ha^−1^ grain yield when compared to the control (*p* = 0.006) at the foliar Mn location. This trend may hold true for the other micronutrients but was not confirmed in this study. Overall, the 1X rate of 0.73 kg Mn ha^−1^ had greater effect on grain yield than did the 2× rate of 1.46 kg Mn ha^−1^. Planned selected contrasts confirmed that the 1× rate had significantly greater effect (*p* = 0.0008) on grain yield than the 2× rate and two separate applications of the 1× rate (i.e., 0.73 kg Mn ha^−1^) and had significantly greater effect (*p* = 0.005) on yield than one application at the 2× rate (i.e., 1.46 kg Mn ha^−1^). Inversely, when there is excess soil supply of the foliar-applied micronutrient, such as in the case of the foliar Zn only location (Winside), there may be yield reduction. This is supported by the significant 4.5%, 0.62 Mg ha^−1^ yield decrease as compared to the control (*p* = 0.03). Mills et al. [26] reported that maize prior to tassel has a Zn leaf concentration sufficiency range of 15–60 mg Zn kg^−1^. At the Zn only location, the split application of 0.84 kg Zn ha^−1^ at V11 and V15 significantly increased the leaf Zn concentration from the control from 24.7 at V14 and 27.3 mg kg^−1^ at V17 to beyond the upper limit of the sufficiency range to 62.0 mg kg^−1^ (*p* < 0.0001) at V14 and 91.3 mg kg^−1^ (*p* < 0.0001) at V17 (Table 4c and Figure 2g), which is consistent with Stewart et al. [37]. Though maize is relatively tolerant to high levels of soil Zn, maize can experience Zn toxicity [38]. Takkar and Mann [38] report leaf tissue Zn above 81.0 kg mg^−1^ can cause grain yield reduction. This threshold was crossed by this treatment likely causing the significant yield reduction.

### 3.3. Foliar Micronutrient Uptake, Partitioning, and Translocation

**Figure 3:**
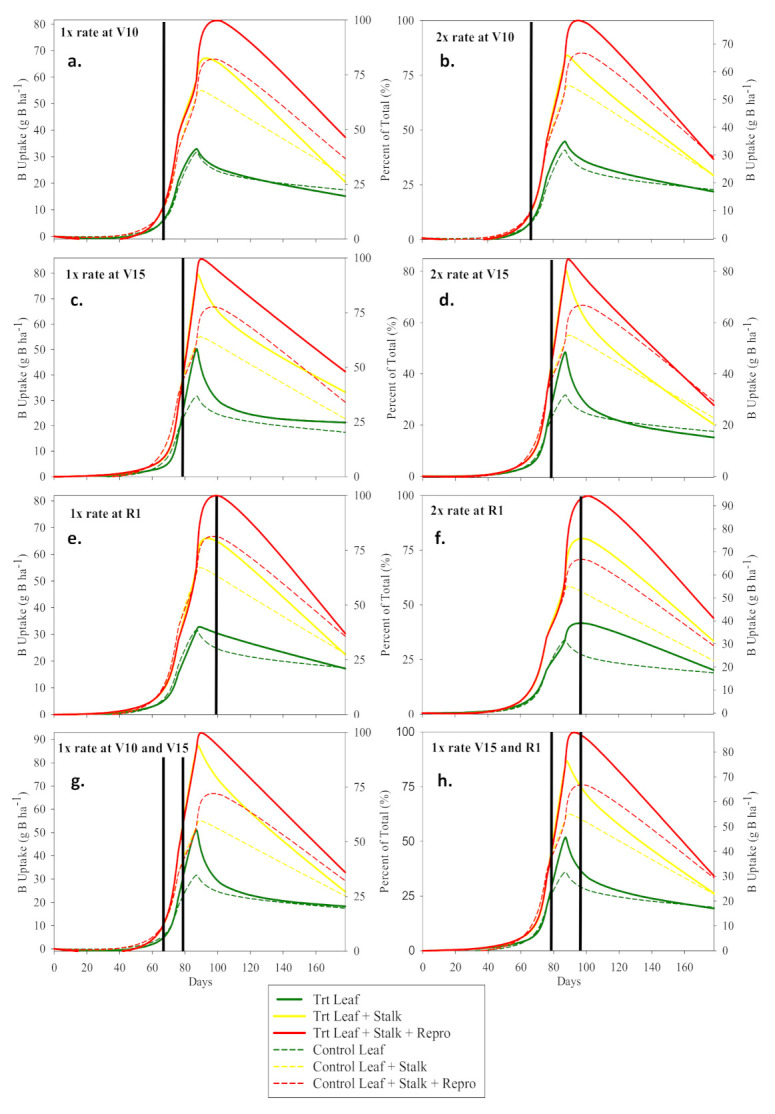
B uptake (g B ha^−1^) and partitioning graphs. Solid and dashed lines represent foliar B treated plots and control plot, respectively. Solid vertical lines represent the time of application expressed as days after sowing. (**a**–**h**) corresponds with the treatments.

**Figure 4:**
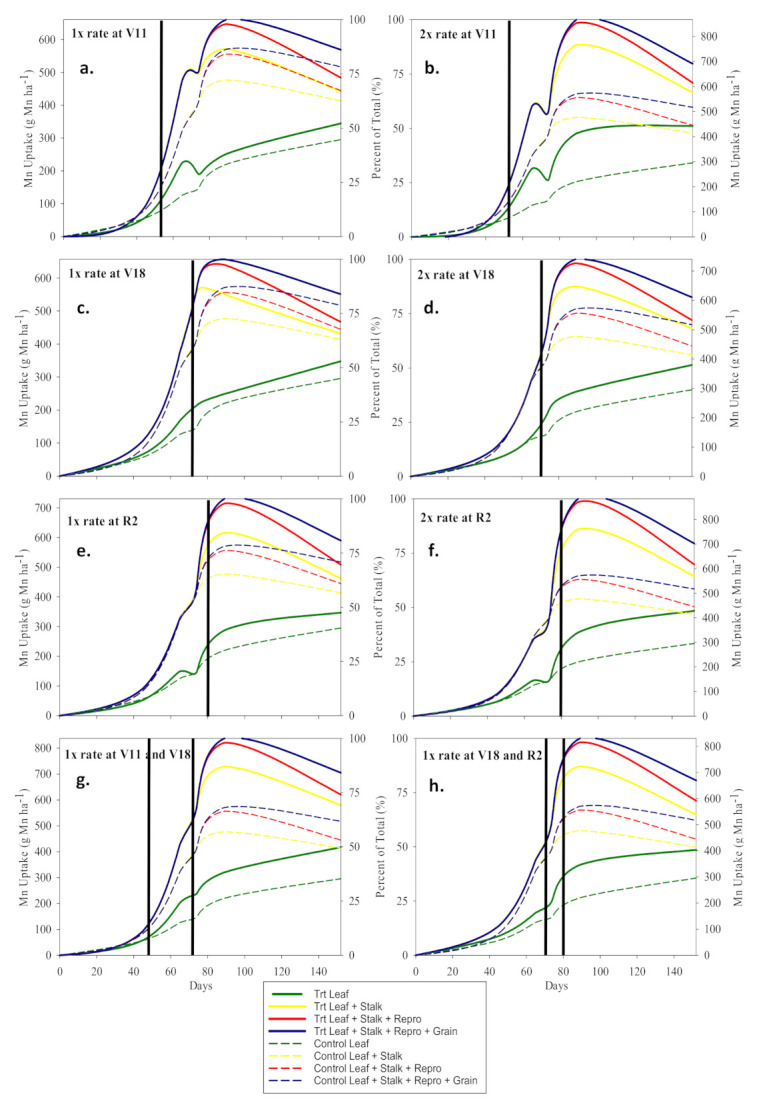
Mn uptake (g Mn ha^−1^) and partitioning graphs. Solid and dashed lines represent foliar Mn treated plots and control plot, respectively. Solid vertical lines represent the time of application expressed as days after sowing. (**a**–**h**) corresponds with the treatments.

Nutrient concentrations and uptake were measured in leaf and stalk prior to VT, leaf, stalk, and reproductive tissue at VT, and leaf, stalk, reproductive tissue, and grain during reproductive stages and is described as such in Table 4a–e and Table 5a–e. Overall, foliar applications of B, Mn, and Zn were effective at increasing their respective micronutrient concentration and uptake in leaf, stalk, reproductive tissues, and immature grain for Mn and Zn throughout the growing season when applied alone (Table 4a–d and Table 5a–c, and Figure 2, Figure 3 and Figure 4). The control B uptake and partitioning was consistent with the B uptake and partitioning reported by Bender et al. [1] though grain B was not detected at 3 mg kg^−1^ detection limit. Partitioned plant sampling following foliar B treatments at rates of both 0.14 kg B ha^−1^ and 0.28 kg B ha^−1^ and at all application times showed significant increase (*p* < 0.05) in B uptake and B concentration in leaf, stalk, and reproductive tissues; however, for all treatments by R6 leaf, stalk, and total B uptake had declined and were not different than the control at *p* < 0.05 (Table 5a and Figure 3). These data are evidence of no additional uptake and mobilization and the likely wash-off of late season (i.e., V15 and R1) foliar applications of B. This was not the case for early season applications of B. Foliar application of B at V10 increased B uptake and mobilization. The T1R1 foliar B application (0.14 kg B ha^−1^ applied at V10) increased the R6 B uptake to the reproductive tissues as compared to the control by 10.3 g B ha^−1^ (*p* = 0.001) which was due to a 3.7 mg B kg^−1^ concentration increase (*p* = 0.0005) (Table 4a and Table 5a). These data suggest that earlier applications of B have greater penetration and mobility as compared to late foliar B applications. Bender et al. [1] reported that stored B in leaf tissue appears to serve as a source of mobilized B to reproductive tissues which is further evident in these data (Figure 3a). Though B is usually considered relatively immobile in cell wall components [39], our data support previous claims that there is a brief period leading up to VT of B mobilization from the leaf tissue to reproductive tissues (Table 5a). V10 or earlier is likely an important target growth stage for foliar B application and may be more successful at inducing grain yield response under more B deficient scenarios.

Foliar Mn and Zn studies had similar effects on their respective nutrient uptake and concentration in stalk and leaf tissue and are therefore discussed together for leaf and stalk components. Across all application stages, the 2× rate (i.e., 1.46 kg Mn ha^−1^ and 0.84 kg Zn ha^−1^) foliar applications of Mn and Zn significantly increased (*p* < 0.05) the R6 total Mn and Zn uptake as compared to the control (Table 5b,c). The 1× rate was not significant. Further, analysis of R6 total plant tissue increases in Zn and Mn uptake due to foliar applications reveals that the foliar Zn and Mn stayed in the leaves and had limited mobility out of the leaves as evident by leaf tissue being the only organs to maintain significant levels (*p* < 0.05) of Zn and Mn uptake at R6 as compared to the control (Table 5b,c). The significant increase in total Zn uptake was largely due to increases in concentration and not biomass, whereas significant increase in total Mn uptake was due to both an increase in concentration and biomass (Table 5b,c and Table 6). It can be theorized that Zn was not limiting, unlike Mn, since the increase in Zn concentration was not associated with an increase in biomass. Unlike Zn, foliar applications of Mn had infrequent significant effect on Mn uptake and concentration in reproductive tissues and grain (Table 4b,c and Table 5b,c). However, the significant increase in reproductive tissue Mn uptake was associated with the only significant increase in maize grain yield. Foliar Zn had greater effect on reproductive tissues and grain than did foliar Mn, especially late season applications. The V15 and R1 applications of foliar Zn increased R2 grain concentration by as much as 10.5 mg Zn kg^−1^ (*p* < 0.0001) and reproductive tissues Zn concentration by as much as 18.8 mg Zn kg^−1^ (*p* < 0.0001) as compared to the control (Table 5c).

Foliar applications of Zn followed a similar trend as Mn uptake in leaf and stalk tissue; however, Zn uptake and mobilization differed from Mn uptake and mobilization late in the growing season from VT to R6 (Figure 2(ex. a) and 2a) (Figure 4). During the reproductive stages (R1–R6), the control Mn uptake plateaued sharply whereas Zn uptake continued to increase and partition to the grain which follows the same trend reported by Bender et al. [1]. The foliar Zn applications did not significantly increase Zn uptake in the R6 grain and reproductive tissues under these growing conditions as was previously reported (Table 5c) [40]. The foliar Mn treated plots had large reductions in Mn uptake during reproductive stages that can be attributed to wash-off of foliar Mn from the leaf surface which was not assimilated during the reproductive stages (Figure 2a). These data provide strong evidence that Mn applications after vegetative growth stages will likely be of no benefit. The sharp Mn uptake plateau during the reproductive growth stages in the control plots follows the same trend reported by Bender et al. [1].

Though no yield response was observed due to reproductive stage applications of Zn under these conditions, reproductive foliar treatments of Zn can be theorized as having potential to affect grain yield. Zn uptake also had foliar Zn wash-off during vegetative growth stages, as evident by in-season spikes in treatment Zn uptake followed by a decrease (Figure 2a), but had non-significant wash-off (i.e., no increase followed by a decrease in Zn uptake following a foliar treatment) for foliar Zn applications applied to maize at reproductive growth stages. This was possibly due to more rapid assimilation during high demand reproductive stages (Figure 2(ex. e.)). At R6, both Mn and Zn uptake significantly increased due to all foliar rate and time treatments. These data suggest that the Zn and Mn, applied to the leaf surface, stayed in the leaf throughout the entirety of the growing season to R6.

**Figure 5:**
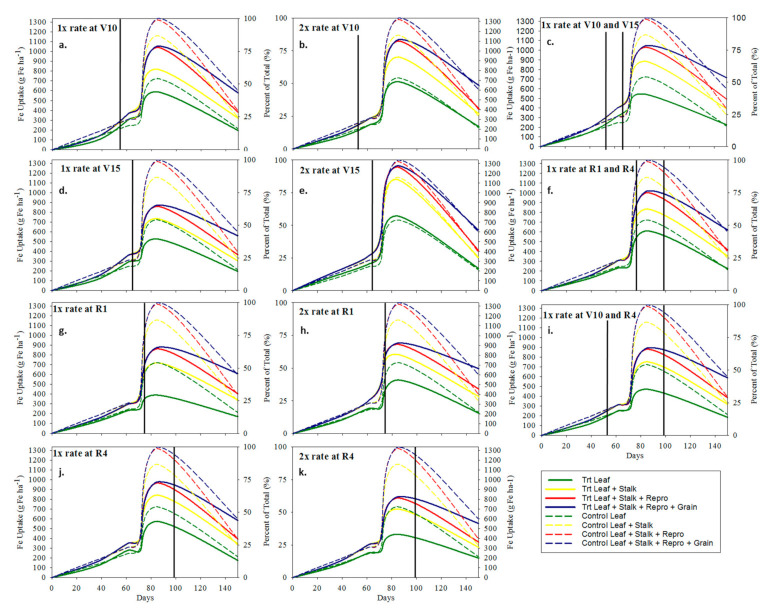
Fe uptake (g Fe ha^−1^) and partitioning graphs. Solid and dashed lines represent foliar Fe/Zn treated plots and control plot, respectively. Solid vertical lines represent the time of application expressed as days after sowing. (**a**–**k**) corresponds with the treatments.

**Figure 6:**
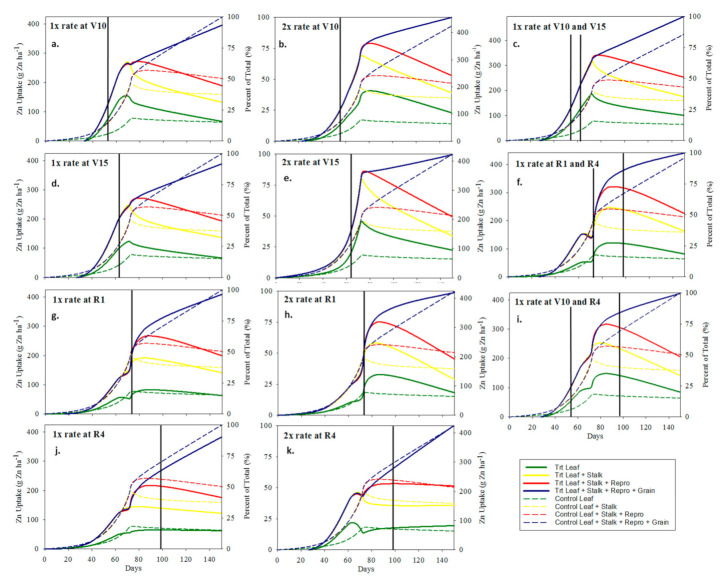
Zn uptake (g Zn ha^−1^) and partitioning graphs. Solid and dashed lines represent foliar Fe/Zn treated plots and control plot, respectively. Solid vertical lines represent the time of application expressed as days after sowing. (**a**–**k**) corresponds with the treatments.

The combined applications of Fe and Zn caused significant suppression of Fe uptake in both leaf and stalk tissues (Table 5e and Figure 5a). The suppression of Fe due to the combined foliar application of Fe and Zn highlights the well-documented Zn-Fe-Mn antagonism as previously documented in maize by Warnock [41]. Zn uptake was not suppressed due to the combined foliar application of Fe and Zn and had similar uptake properties as observed at the foliar Zn only location (Table 5c–e, and Figure 2a and Figure 6a). Additional investigation confirms that there was no reduction in biomass as compared to the control driving the reduction in Fe uptake, rather the reduction in Fe uptake was driven by a reduction in plant tissue concentrations of Fe in treated plots (Table 4e and Table 7). Bender et al. [1] also reported significant reduction in Fe uptake after VT and they theorized that this was due to pollen and silks (styles) shed which contains Fe [42] being greater than the uptake rate. Additionally, foliar Fe applications have also been shown to depress the plant’s Fe stress mechanisms by preventing the increase in the Fe-reducing capacity of the roots that would normally occur during Fe deficiency [41]. Investigation of the biomass data from this location indicates that the reduction in Fe uptake was not due to biomass reduction (i.e., no treatment biomass was significantly different (*p* < 0.05) than the control biomass) (Table 7).

### 3.4. Recovery Efficiency of Foliar Micronutrients

Foliar applications of Mn and Zn had similar but slightly higher ANR than reported soil applied Mn and Zn ANR [18]. Mortvedt [18] reports soil Mn and Zn ANR to range from 5–10%. For all locations, there were no ANR treatment main effects at *p* < 0.05. There was a consistent trend for both Zn and Mn ANR at the 2X rate to always have a greater ANR than the 1X rate for treatments applied at the same growth stage (Tables S2 and S3). Since there were no differences between treatment rates, ANR for all treatment rates were combined. Foliar Mn, Zn, and B only application had ANR least square means of 9.5, 16.9, and 2.5%, respectively with standard errors of 3.7, 9.6, and 2.9, respectively. The foliar application of a mix of Fe and Zn had negative ANR indicating suppression of Fe and Zn uptake which is consistent with the findings of Römheld and Marschner [43], who found reduced Fe uptake in grasses due to foliar Fe. At the foliar Fe/Zn location, the least square mean ANR for Zn was −1.3% with a standard error of 6.1 and the ANR of Fe was −9.1% with a standard error of 20.5 (Table 6).

The low ANR of each of the applied micronutrients implies that most of the foliar application was either sprayed directly onto the soil or was washed-off the leaf surface. Figure 2a–Figure 5a all show a spike in their respective nutrient uptake immediately following foliar application; however, by the time of the next foliage sampling, there was a large reduction in the nutrient uptake likely due to wash-off of the treatment from the leaf surface. All foliar treatments were on the leaf surface for at least 24 h prior to an irrigation or rain event. Our data also indicates that in some cases there may be suppression of the applied micronutrient under conditions of micronutrient toxicity (i.e., as in the case of Fe uptake following an Fe/Zn treatment under excessive Fe tissue concentrations), the actual amount of foliar-applied micronutrient being recovered by maize may be higher but cause suppression of the applied micronutrient in the plant tissue. For example, the maize plants may have recovered a higher percent of the foliar-applied micronutrient, but the treatment may have suppressed soil uptake thereby reducing the total amount of the micronutrient in the plant tissue. Though the pathway is not clear, it could be speculated that sink plant organs that have excessive levels of the nutrient could signal to the root uptake cells to reduce the corresponding nutrient’s uptake. Whether most of the foliar treatment is falling to the soil or being recovered by the plant and suppressing soil uptake remains unresolved. What these data show is that foliar applications of micronutrients have a low ANR and the overall micronutrient status of the plant tissue per unit of applied micronutrient is usually less than 20% which similar but slightly higher than soil applications [17,18]. However, this small increase in ANR may be critical if maize micronutrient status is near the critical level at a critical growth stage.

## 4. Conclusions

Nebraska soils are generally micronutrient sufficient [44]. Stewart et al. [45] have highlighted the effect of micronutrient foliar application on maize yield. However, through this study we identified how the combination of these micronutrients and their application during peak demand influence uptake, translocation, and partitioning and the influence on maize yield. Though yield increases were observed under specific conditions, under field conditions, foliar applications of Zn, B, or combined Zn and Fe treatments would be challenging to be applied in a way that returns predictable grain yield increases. There were some evidence of significant yield decreases even when concentrations of the applied nutrient were above its sufficiency range, as was found in the case of the Zn only and Zn/Fe locations. Under conditions of acute reductions in Mn or Fe availability, applications of foliar Mn or Fe during those specific periods may increase grain yield.

Of greatest interest, these data indicate that foliar applications, applied at any growth stage of maize with confirmed Fe deficiency increased grain yield significantly. Though there was limited yield response to foliar B, Mn, and Zn under our study conditions, these data provide evidence for target growth stages to increase micronutrient uptake and mobilization of the applied micronutrient to tissues with physiological demand. There should also be caution when applying mixes of foliar micronutrients as there can be significant reductions in micronutrient uptake as evident by Fe suppression due to the combined application of Fe and Zn. Applying micronutrients to locations with sufficient to high levels of the applied micronutrient may also have significant yield reduction. Further, ANR for individually applied foliar micronutrients were much less than soil recovery efficiencies (i.e., ANR were largely less than 25%). This study design could not exclusively define that all foliar applied micronutrients were taken up through areal plant tissues as opposed to reaching the soil and uptake through the roots. This fate of foliar applied nutrients should be further studied. These data could be used to calculate application rates with specific goals of increasing micronutrient concentrations in plant tissue from below critical values to above critical values. These results can also be used to guide the use of foliar micronutrients for agronomic biofortification of maize grain. In conclusion, this study showed that foliar applications of B, Mn, Zn, and Fe had limited effect on grain yield in regions with soils and conditions like those of this study unless there is a confirmed micronutrient deficiency. A summary of our recommendations is provided in Table S3.

## Figures and Tables

**Table 1:** Background information and cultural practices for the five foliar micronutrient experimental locations.

Year	Treatment	Coordinates		Avg Rainfall † (mm)	GDD ‡ (Season)	Till §	Irrigation (Y/N)	Environmental Factors	Hybrid	Planting Date	Harvesting Date
		Lattitude	Longitude								
2014	Boron	41.83	−96.49	646	3187	CT	Y	-	Croplan 6274	25 April	8 Nov.
2014	Manganese	42.17	−97.08	630	3432	CT	Y	Standing water at 7/6 (V12) (2 weeks standing water)	GH G14R38	7 May	7 Nov.
2014	Zinc	42.15	−97.05	608	3138	NT	N	Hail 7/6 (V9) (5–10% yield reduction)	Pioneer 1625	20 May	18 Nov.
2014	Iron & Zinc	41.97	−97.74	608	3138	NT	N	Hail 7/6 (V9) (5–10% yield reduction)	Pioneer 1625	20 May	9 Nov.
2015	Iron	40.55	−101.69	463	3374	CT	Y	-	Pioneer 1151	25 April	20 Nov.

† Observed and average (2005–2014) rainfall during growing season (from April-October). ‡ GDD (Growing Degree Days); observed during the growing season (from April-October) (GDD = Daily avaerage tempearture −10 °C [Base temperature for maize]) [25]. § Till = tillage system including conventional tillage consisting of disk or chisel plow tillage (CT) or no-till (NT).

**Table 2:** Background leaf (V5–6) and soil characteristics for the study sites.

Site	Applied Nutrient	Sampling Stage	Leaf Analysis				Soil Analysis (0–20 cm)							
			Mn	B	Zn	Fe	Texture †	SOM	CEC	pH	Mn	B	Zn	Fe
			(mg kg^−1^)					(%)	meq./100 g	−log (H^+^)	(mg kg^−1^)			
1	B	V5	74.3	6.0	22.5	270.3	SiCL	2.8	18.2	5.6	20.3	0.65	2.43	100.8
2	Mn	V5	62.0	8.0	20.0	265.5	SiCL	3.2	27.1	6.4	22	0.88	1.68	70.5
3	Zn	V5	130.5	6.8	22.3	308.3	SiCL	3.2	27.1	5.1	37.3	0.73	0.9	53.5
4	Zn & Fe	V5	180.0	9.8	20.0	403.3	SiCL	3.6	29.4	5.0	44.3	1	1.43	109.5
5	Fe	V6	129.3	12.4	102.0	181.1	L	2.4	16.2	7.5	1.9	0.8	4.3	4.9
	Critical Level ‡		15.0	4.0	15.0	10.0	-	-	-	-	2.0	0.5	0.75	4.5

† Soil texture classes include silty clay loam (SiCL), and loams (L). ‡ Leaf analysis critical levels are from Mills and Jones (prior to tassel) and soil analysis critical levels are from Wortman et al. (2009) and Ward (2015).

**Table 3:** Multiple comparison test of LSmean yields (Mg ha^−1^) comparing foliar-applied micronutrient treatment effects applied at different rates and growth stages with the control using Dunnett’s Test.

Foliar Micronutrient Treatment Locations					
2014					2015
Treatments	Boron	Manganese	Zinc Only	Fe & Zn	Fe Only
Control	14.91(0.20) †	7.90(0.38)	13.70(0.20)	12.70(0.28)	14.22(0.68)
T1R1 ‡	14.56	8.83 +	13.55	12.57	14.69
T1R2	14.71	7.17	13.49	12.69	16.29 *
T2R1	14.68	9.42 **	13.21+	12.54	14.61
T2R2	14.62	7.90	13.41	12.37	15.64
T1R1 and T2R1	15.00	8.66	13.08 **	11.63 +	16.24 *
T3R1	14.37 +	8.03	13.49	12.69	16.06 +
T3R2	14.68	7.90	13.26 +	12.26	16.14 *
T2R1 and T3R1	14.54	8.72	13.30	-	15.46
T4R1	-	-	-	13.48 +	-
T4R2	-	-	-	12.71	-
T1R1 and T4R1	-	-	-	12.86	-
T3R1 and T4R1	-	-	-	12.47	-

† Least square mean yield (Mg ha^−1^) followed by (SE for all values in the same column) and by significant F test: Not Significant >0.10; + >0.05; * >0.01; ** >0.001; *** <0.001. ‡ T = Time of foliar application (1: early (V6–11), 2: middle (V15–18), 3: 4: late (R1–4)), R = Treatment rate (rate 1: lower level of industry recommendation & rate 2: upper level of industry recommendation).

**Table 4:** (**a**–**e**). Multiple comparison test of LSmeans of nutrient concentrations (mg kg^−1^) in partitioned plant tissues at various stages comparing treatment effects with control.

(a)													
Location	Component and Stages	Control		T1R1	T1R2	T2R1	T2R2	T1R1 & T2R1	T3R1		T3R2	T2R1 & T3R1	
**B**	**Leaf**												
	V6	3.3(0.7) †,‡		3.3 ‡	3.0 ‡	3.5 ‡	4.0 ‡	3.0 ‡	4.8 ‡		5.0 + ‡	4.5 ‡	
	V13	6.8(0.8) ‡		7.5 §	8.3 +§	6.5 ‡	7.3 ‡	8.2 +§	6.0 ‡		7.0 ‡	7.0 ‡	
	V17	8.8(1.7) ‡		9.7	10.3	14.0 **§	14.7 ***§	14.5 **§	8.8 ‡		9.2 ‡	14.0 **§	
	R2	7.3(0.8) ‡		7.2	8.2	8.7 +	8.0	8.3	8.8 +§		12.2 ***§	9.0 *§	
	R6	4.8(0.6) ‡		4.5	4.8	6.0 *	4.5	5.0	5.0		5.7	4.8	
	**Stalk**												
	V6	5.5(0.4) ‡		4.8 ‡	5.5 ‡	5.0 ‡	5.8 ‡	5.3 ‡	5.0 ‡		4.5 +‡	5.0 ‡	
	V13	5.2(0.9) ‡		6.0 §	5.7 §	4.8 ‡	5.3 ‡	7.0 +§	4.8 ‡		5.7 ‡	4.2 ‡	
	V17	3.8(0.8) ‡		5.0	5.8 *	5.2 §	6.7 **§	6.5 **§	4.3 ‡		4.7 ‡	5.8 *§	
	R2	4.2(0.6) ‡		5.7 *	4.8	5.5 *	4.8	6.0 **	5.3 +§		5.8 **§	4.8 §	
	R6	1.0(1.0) ‡		1.0	1.2	2.3	1.0	1.2	1.0		2.5	1.2	
	**Reproductive**												
	R2	6.0(2.2) ‡		5.5	6.5	6.2	6.0	5.3	6.3 §		7.8 §	10.3 +§	
	R6	2.3(1.0) ‡		6.0 ***	2.2	2.7	2.7	2.8	3.0		3.3	2.5	
**(b)**													
**Location**	**Component and Stages**	**Control**		**T1R1**	**T1R2**	**T2R1**	**T2R2**	**T1R1 & T2R1**	**T3R1**		**T3R2**	**T2R1 & T3R1**	
**Mn**	**Leaf**												
	V7	76.8(6.4) ‡		72.0 ‡	76.3 ‡	75.3 ‡	68.3 ‡	69.3 ‡	67.8 ‡		74.3 ‡	83.8 ‡	
	V15	65.7(13.8) ‡		118.5 ***§	141.3 ***§	79.3 ‡	68.8 ‡	101.7 **§	69.0 ‡		78.3 ‡	77.2 ‡	
	VT	83.5(10.4) ‡		102.7 +	123.8 ***	108.7 *§,¶	118.2 **§	129.7 ***§	79.8 ‡		76.8 ‡	115.7 **§	
	R3	104.7(11.8) ‡		116.3	174.2 ***	106.7	133.7 *	136.5 **	132.7 *§		161.2 ***§	153.2 ***§	
	R6	150.0(9.9) ‡		163.7	203.8 ***	159.7	182.2 **	184.2 ***	177.3 **		206.8 ***	195.2 ***	
	**Stalk**												
	V7	75.5(6.5) ‡		79.8 ‡	90.0 ‡	85.0 ‡	77.3 ‡	77.0 ‡	82.3 ‡		72.5 ‡	84.0 ‡	
	V15	77.7(7.5) ‡		86.7 §	101.0 **§	68.7 ‡	76.2 ‡	83.3 §	75.8 ‡		70.2 ‡	72.3 ‡	
	VT	87.5(6.6) ‡		81.3	80.2	84.7 §,¶	87.8 §	87.3 §	97.3 ‡		76.7 ‡	86.5 §	
	R3	78.2(7.8) ‡		86.0	93.5+	80.3	104.3 **	99.7 **	94.7 *§		120.8 ***§	101.5 **§	
	R6	40.8(6.0) ‡		34.3	43.7	27.3 *	42.8	49.2	43.2		46.7	43.3	
	**Reproductive**												
	VT	79.3(13.6) ‡		88.8	83.3	114.0 *§,¶	89.5 §	94.3 §	86.0 ‡		87.7 ‡	89.0§	
	R3	23.3(5.4) ‡		21.3	25.7	25.0	24.0	26.7	31.5 §		36.5 *§	26.2 §	
	R6	15.2(5.2) ‡		18.7	17.7	15.8	14.0	18.0	22.8		22.0	21.3	
	**Grain**												
	R3	15.9(1.8) ‡		13.7	12.0 *	12.3 *	13.9	13.7	15.3 §		13.6 §	14.1 §	
	R6	5.8(0.3) ‡		6.0	6.1	5.9	6.1	5.7	5.9		6.3 +	5.8	
**(c)**													
**Location**	**Component and Stages**	**Control**		**T1R1**	**T1R2**	**T2R1**	**T2R2**	**T1R1 & T2R1**	**T3R1**		**T3R2**	**T2R1 & T3R1**	
**Zn only**	**Leaf**												
	V6	26.3(0.9 )‡		27.5 ‡	26.5 ‡	25.0 ‡	25.8 ‡	25.3 ‡	26.8 ‡		27.8 ‡	27.8 ‡	
	V14	24.7(5.9) ‡		71.8 ***§	101.0 ***§	22.7 ‡	21.7 ‡	62.0 ***§,#	24.2 ‡		22.0 ‡	22.5 ‡	
	V17	27.3(9.5) ‡		58.2 **	85.5 ***	78.2 ***§	108.8 ***§	91.3 ***§,#	27.3 ‡		28.5 ‡	73.3 ***§	
	R2	29.2(22.0) ‡		49.0	80.0 *	54.5	74.2 *	123.2 ***	63.0 §		84.0 *§	88.5 **§	
	R6	34.2(7.6) ‡		47.8 +	73.5 ***	57.8**	73.3 ***	68.0 ***	50.7 *		77.2 ***	71.2 ***	
	**Stalk**												
	V6	55.3(3.2) ‡		52.0 ‡	46.5 +‡	50.0 ‡	53.3 ‡	47.5 ‡	60.0 ‡		53.3 ‡	55.5 ‡	
	V14	26.8(5.0) ‡		39.3 *§	73.3 ***§	37.5 *‡	32.3 ‡	42.7 **§,#	29.5 ‡		30.5 ‡	35. 3 ‡	
	V17	23.7(3.0) ‡		27.0	45.8 ***	36.2 ***§	55.5 ***§	44.0 ***§,#	24.7 ‡		24.7 ‡	35.2 ***§	
	R2	19.0(3.5) ‡		26.0 +	31.5 ***	27.3 *	41.0 ***	36.0 ***	27.5 *§		44.0 ***§	41.0 ***§	
	R6	27.0(6.3) ‡		25.8	28.5	29.8	37.8 +	30.3	31.3		41.2 *	37.0	
	**Reproductive**												
	R2	26.5(2.8) ‡		28.7	30.3	29.3	34.7 **	32.8 *	38.2 ***§		45.3 ***§	41.2 ***§	
	R6	34.7(5.9) ‡		34.3	43.0	30.0	35.2	34.2	30.3		39.8	39.7	
	**Grain**												
	R2	47.5(2.3) ‡		48.8	52.1 +	50.9	49.9	52.6 *	55.2 **§		53.9 **§	58.0 ***§	
	R6	19.3(0.6) ‡		19.8	20.3	18.6	19.4	19.7	20.5+		19.9	20.1	
**(d)**													
**Location**	**Component and Stages**	**Control**	**T1R1**	**T1R2**	**T2R1**	**T2R2**	**T1R1 & T2R1**	**T3R1**	**T3R2**	**T4R1**	**T4R2**	**T1R1 & T4R1**	**T3R1 & T4R1**
**Fe/Zn** **(Zn** **Values)**	**Leaf**												
	V6	25.8(0.9) ‡	25.5 ‡	25.5 ‡	26.0 ‡	28.5 *‡	28.0 +‡	25.0 ‡	26.5 ‡	26.0 ‡	27.0 ‡	24.8 ‡	24.8 ‡
	V14	22.3(13.6) ‡	74.8 **§	53.8 §	51.5 ‡	40.0 ‡	68.5 *§	30.5 ‡	24.0 ‡	29.0 ‡	51.0 ‡	50.8 §	27.3 ‡
	V17	31.3(6.1) ‡	63.5 ***	65.0 ***	56.5 **§	80.0 ***§	80.3 ***§	26.3 ‡	27.8 ‡	24.8 ‡	25.3 ‡	49.3 *	25.0 ‡
	R2	34.5(7.8) ‡	55.5 +	86.0 ***	49.5	73.8 **	69.3 **	40.0 §	66.8 **§	31.3 ‡	34.0 ‡	71.0 **	55.5 +§
	R6	29.5(2.7) ‡	32.3	50.5 ***	32.0	43.3 ***	44.8 ***	30.3	36.8 +	30.3 §	39.3 *§	40.8 **§	37.5 *§
	**Stalk**												
	V6	51.0(3.2) ‡	44.8 ‡	50.3 ‡	46.0 ‡	62.3 *‡	52.5 ‡	42.4 +‡	54.0 ‡	49.0 ‡	52.8 ‡	44.8 ‡	54.3 ‡
	V14	35.3(4.4) ‡	52.3 **§	51.3 *§	50.8 *‡	37.3 ‡	44.0 §	32.5 ‡	29.5 ‡	40.0 ‡	45.8 +‡	48.3 *§	48.5 *‡
	V17	26.3(3.3) ‡	29.0	38.8 **	31.3 §	31.0 §	31.0 §	26.3 ‡	23.0 ‡	23.8 ‡	28.3 ‡	34.3 +	21.5 ‡
	R2	21.0(2.8) ‡	18.8	22.5	19.8	24.8	23.8	23.0 §	23.5 §	18.0 ‡	17.8 ‡	22.5	25.3 §
	R6	18.8(2.8) ‡	15.0	15.8	16.5	12.0 +	15.5	15.8	10.8	12.5 §	16.8 §	12.8 §	15.5 §
	**Reproductive**												
	R2	29.5(2.8) ‡	31.3	31.8	37.0 +	32.0	35.3	36.8 +§	37.3 +§	36.8 +‡	31.0 ‡	34.0	35.3 §
	R6	27.8(2.8) ‡	28.0	32.3	28.3	31.8	34.8 +	28.3	31.3	26.5 §	32.3 §	30.3 §	29.8 §
	**Grain**												
	R2	48.4(5.9) ‡	65.2+	56.1	55.1	61.9	68.7 *	77.2 **§	66.8 *§	52.3 ‡	58.2 ‡	63.5 +	66.2 *§
	R6	19.4(1.0) ‡	18.5	19.3	17.8	19.5	20.8	18.5	18.9	18.3 §	18.4 §	18.7 §	19.5 §
**(e)**													
**Location**	**Component and Stages**	**Control**	**T1R1**	**T1R2**	**T2R1**	**T2R2**	**T1R1 & T2R1**	**T3R1**	**T3R2**	**T4R1**	**T4R2**	**T1R1 & T4R1**	**T3R1 & T4R1**
**Fe/Zn** **(Fe Values)**	**Leaf**												
	V6	305.0(18.9 )‡	281.5 ‡	284.3 ‡	265.3 ‡	321.8 ‡	306.5 ‡	275.8 ‡	316.8 ‡	286.5 ‡	282.0 ‡	275.8 ‡	282.0 ‡
	V14	133.3(17.0) ‡	170.0 §	142.0 §	159.8 ‡	152.3 ‡	171.5 §	128.5 ‡	138.5 ‡	162.0 ‡	139.8 ‡	143.3 §	119.5 ‡
	V17	143.0(13.5) ‡	163.0	163.0	153.8 §	183.3 *§	184.0 *§	131.8 ‡	120.3 ‡	132.3 ‡	116.9 ‡	137.8	124.3 ‡
	R2	330.3(38.2) ‡	277.8	319.3	253.0	350.3	248.5	189.8 **§	264.3 §	274.5 ‡	213.3 *‡	197.0 *	286.8 §
	R6	98.5(7.5) ‡	94.3	107.3	94.3	103.3	99.3	82.5	98.8	84.8 §	94.5 §	89.0 §	103.5 §
	**Stalk**												
	V6	177.5(25.9) ‡	187.3 ‡	159.5 ‡	157.0 ‡	166.8 ‡	194.5 ‡	152.3 ‡	211.3 ‡	176.0 ‡	160.3 ‡	194.3 ‡	158.8 ‡
	V14	33.5(5.3) ‡	34.5 §	38.0 §	35.0 ‡	42.5 ‡	40.3 §	32.0 ‡	39.8 ‡	40.5 ‡	44.5 ‡	34.5 §	37.5 ‡
	V17	37.3(10.8) ‡	35.0	32.3	32.3 §	50.3 §	34.5 §	40.8 ‡	59.3 ‡	40.5 ‡	38 ‡	32.8	38.5 ‡
	R2	86.8(12.7) ‡	50.0 *	52.3 +	44.0 *	74.8	72.3	69.0 §	56.8 §	58.0 ‡	53.8 +‡	63.0	46.0 *§
	R6	23.5(5.5) ‡	29.5	28.3	24.8	28.0	33.3	33.5	40.5 *	36 §	26.8 §	30 §	25.8 §
	**Reproductive**												
	R2	73.3(15.7) ‡	110.8	76.5	61.5	58.0	74.3	69.8 §	52.8 §	61.3 ‡	51.0 ‡	64.5	80.3 §
	R6	33.8(6.8) ‡	30.0	27.8	31.0	33.5	42.8	30.5	34.8	23.8 §	26.3 §	31.8 §	29.8 §
	**Grain**												
	R2	33.5(5.0) ‡	42.1	38.3	36.0	47.5 *	45.0	49.5 *§	42.8 §	36.0 ‡	44.9 ‡	42.3	43.1 §
	R6	19.0(1.1) ‡	17.6	21.6	17.8	19.2	19.4	18.3	18.0	17.0 §	16.9 §	17.6 §	18.7 §

† Least square mean plant nutrient concentration (mg kg^−1^) followed by (SE for all values in the same row) and significant F test: Not Significant >0.10; + >0.05; * >0.01; **> 0.001; ***< 0.001. ‡ No foliar treatment had been applied at this stage. § First sampling following foliar treatment. ¶ First sampling following foliar treatment for plots with significant increase on grain yield. # First sampling following foliar treatment for plots with significant decrease on grain yield.

**Table 5:** (**a**–**e**). Multiple comparison test of LSmeans of nutrient quantity (g ha^−1^) in partitioned plant tissues at various stages comparing treatment effects with control.

(a)										
Location	Component and Stages	Control	T1R1	T1R2	T2R1	T2R2	T1R1 & T2R1	T3R1	T3R2	T2R1 & T3R1
**B**	**Leaf**									
	V6	2.7(0.5) †, ‡	2.2 ‡	2.2 ‡	2.5 ‡	2.7 ‡	1.8 ‡	2.8 ‡	3.3 ‡	3.3 ‡
	V13	18.5(2.3) ‡	21.0 §	21.7 §	16.9 ‡	19.6 ‡	22.2+ §	15.4 ‡	18.8 ‡	18.4 ‡
	V17	31.7(6.3) ‡	32.9	34.9	50.3 ** §	48.5 ** §	51.1 ** §	31.3 ‡	31.3 ‡	45.5 * §
	R2	24.1(2.1) ‡	25.3	27.0	28.9+	26.9	29.0+	30.0* §	38.9 *** §	28.9+ §
	R6	17.5(2.1) ‡	15.1	16.8	21.3+	15.1	18.2	17.2	18.7	17.0
	**Stalk**									
	V6	1.8(0.3) ‡	1.4 ‡	1.7 ‡	1.6 ‡	1.5 ‡	1.6 ‡	2.0 ‡	1.4 ‡	1.5 ‡
	V13	14.0(3.8) ‡	17.1 §	13.1 §	11.2 ‡	13.0 ‡	19.8+ §	12.5 ‡	14.0 ‡	11.7 ‡
	V17	20.9(4.9) ‡	26.1	28.8 +	27.3 §	30.9** §	34.6** §	24.1 ‡	26.0 ‡	28.9 + §
	R2	26.9(3.0) ‡	39.5**	31.4	34.8 +	32.1	42.2**	34.1 + §	36.0* §	32.5 §
	R6	5.3(4.7) ‡	5.4	5.8	11.9	5.1	6.3	5.3	12.6 +	6.1
	**Reproductive**									
	R2	15.2(3.1) ‡	16.4	18.6	16.3	16.3	15.2	17.7 §	18.6 §	22.9 + §
	R6	6.5(2.9) ‡	16.8***	5.9	8.1	7.5	8.4	7.8	9.9	7.0
	**Total**									
	V6	4.5(0.5) ‡	3.6 ‡	3.9 ‡	4.1 ‡	4.1 ‡	3.4 ‡	4.7 ‡	4.7 ‡	4.8 ‡
	V13	32.5(5.0) ‡	38.1 §	34.9 §	28.1 ‡	32.6 ‡	42.0 + §	27.9 ‡	32.8 ‡	30.1 ‡
	V17	52.6(8.2) ‡	59.0	63.7	77.5 ** §	79.4** §	85.7 *** §	55.4 ‡	57.3 ‡	74.4 ** §
	R2	66.2(5.0) ‡	81.2*	77.0	80 +	75.4	86.4 **	81.7* §	93.5 *** §	84.2 * §
	R6	29.3(7.0) ‡	37.3	28.5	41.3 +	27.8	32.9	30.3	41.2 +	30.0
**(b)**										
**Location**	**Component and stages**	**Control**	**T1R1**	**T1R2**	**T2R1**	**T2R2**	**T1R1 & T2R1**	**T3R1**	**T3R2**	**T2R1 & T3R1**
**Mn**	**Leaf**									
	V7	56.7(7.3) ‡	59.2 ‡	58.4 ‡	66.2 ‡	58.1 ‡	58.0 ‡	52.6 ‡	58.4 ‡	73.4 ‡
	V15	125.9(32.7) ‡	225.1 ** §	272.4 *** §	167.3 ‡	135.1 ‡	202.5* §	149.3 ‡	145.5 ‡	162.7 ‡
	VT	147.0(24.6) ‡	189.9 +	227.1 **	213.1 ** §,¶	216.8 ** §	237.6*** §	146.1 ‡	151.1 ‡	210.2 ** §
	R3	219.8(25.0) ‡	251.1	412.5 ***	248.3	288.1 +	335.1**	287.3 + §	326.2** §	346.1 *** §
	R6	295.4(30.9) ‡	344.6 +	442.44 ***	347.4 +	380.1**	416.8***	346.7 +	428.0***	403.4***
	**Stalk**									
	V7	27.3(5.8) ‡	35.4 ‡	38.5 ‡	40.8 ‡	35.8 ‡	35.7 ‡	39.4 ‡	30.1 ‡	38.9 ‡
	V15	197.0(28.1) ‡	251.6 + §	249.0 + §	206.4 ‡	189.7 ‡	225.8 §	178.8 ‡	168.0 ‡	202.1 ‡
	VT	259.4(37.3) ‡	305.7	267.9	339.1* §,¶	293.3 §	325.8 + §	280.1 ‡	264.4 ‡	303.7 §
	R3	257.3(26.4) ‡	320.8 +	353.8**	303.1	359.8**	414.0 ***	329.2 + §	406.4 *** §	377.3 ** §
	R6	118.5(23.2) ‡	97.4	133.5	84.2	123.9	163.1 +	115.7	141.5	134.9
	**Reproductive**									
	VT	5.3(1.4) ‡	6.9	6.2	8.7 ** §,¶	6.4 §	6.5 §	5.7 ‡	7.2 ‡	7.6 + §
	R3	79.3(12.8) ‡	75.8	87.1	88.6	79.0	92.8	98.2 §	103.8 §	92.5 §
	R6	31.1(11.6) ‡	42.1	39.0	35.8	29.3	40.6	45.9	47.8	53.6 +
	**Grain**									
	R3	14.4(1.7) ‡	13.0	13.2	16.5	12.9	18.0	15.5 §	11.7 §	14.7 §
	R6	72.4(6.5) ‡	85.1 +	76.2	83.8 +	77.7	84.8 +	81.3	85.4*	78.0
	**Total**									
	V6	84.0(11.6) ‡	94.6 ‡	96.9 ‡	107.0 ‡	93.9 ‡	93.7 ‡	92.0 ‡	88.5 ‡	112.3 + ‡
	V13	322.9(47.8) ‡	476.7 ** §	521.4 *** §	373.6 ‡	324.8 ‡	428.3 ** §	328.1 ‡	313.5 ‡	364.8 ‡
	V17	411.7(57.0) ‡	502.5	501.0	561.0** §	516.4 + §	569.9 ** §	432.0 ‡	422.7 ‡	521.6 + §
	R2	570.8(52.4) ‡	660.8	845.2***	681.0	739.7*	857.6 ***	730.2 * §	850.6 *** §	830.6*** §
	R6	517.4(52.9) ‡	569.1	691.2**	551.2	611.0 +	705.2 ***	589.5	702.7 ***	669.9 **
**(c)**										
**Location**	**Component and Stages**	**Control**	**T1R1**	**T1R2**	**T2R1**	**T2R2**	**T1R1 & T2R1**	**T3R1**	**T3R2**	**T2R1 & T3R1**
**Zn only**	**Leaf**									
	V6	10.5(0.7) ‡	11.1 ‡	9.9 ‡	9.5 ‡	11.0 ‡	10.4 ‡	10.9 ‡	11.7 ‡	12.7* ‡
	V14	51.9(13.9) ‡	154.5 *** §	213.6 *** §	45.9 ‡	47.2 ‡	123.9*** §,#	53.1 ‡	45.5 ‡	47.6 ‡
	V17	86.6(26.8) ‡	175.0**	227.9 ***	207.4 *** §	298.6 *** §	242.0 *** §,#	75.9 ‡	77.5 ‡	206.8*** §
	R2	74.6(52.9) ‡	125.0	192.3 *	135.5	174.8 +	292.4 ***	152.5 §	209.6* §	213.3** §
	R6	102.1(23.7) ‡	153.1 *	210.9 ***	169.7**	216.1 ***	200.5***	148.5 +	224.3***	213.1 ***
	**Stalk**									
	V6	10.5(1.3) ‡	10.3 ‡	7.3 ‡	8.9 ‡	11.9 ‡	10.2 ‡	11.6 ‡	10.3 ‡	13.8 + ‡
	V14	94.9(36.4) ‡	135.2 §	210.7 ** §	117.9 ‡	98.6 ‡	134.2 §,#	78.0 ‡	89.8 ‡	96.6 ‡
	V17	101.0(15.6) ‡	123.8	192.2 ***	148.0 ** §	244.9 *** §	178.5 *** §,#	109.5 ‡	100.7 ‡	151.0 ** §
	R2	115.0(25.2) ‡	150.3	181.1**	159.0 +	213.8***	200.9**	147.6 §	256.8*** §	234.9*** §
	R6	140.1(36.0) ‡	137.9	136.5	145.4	197.3	164.2	155.5	214.5*	193.8
	**Reproductive**									
	R2	54.6(6.1) ‡	57.1	61.3	58.4	64.2	60.7	70.9** §	91.1*** §	80.4*** §
	R6	82.6(15.0) ‡	81.4	108.9 +	71.2	78.2	75.5	72.6	89.8	87.6
	**Grain**									
	R2	20.0(2.0) ‡	17.7	15.2*	15.3*	17.2	17.6	16.2 + §	20.0 §	20.1 §
	R6	243.2(12.4) ‡	252.3	242.0	232.9	229.1	230.9	265.5 +	246.7	249.2
	**Total**									
	V6	21.1(2.2) ‡	21.4 ‡	14.9* ‡	18.4 ‡	22.9 ‡	20.6 ‡	24.5 ‡	22.0 ‡	26.5* ‡
	V14	146.8(44.5) ‡	289.7* §	424.3 *** §	163.8 ‡	145.9 ‡	258.1** §	131.1 ‡	135.3 ‡	144.1 ‡
	V17	187.7(32.6) ‡	298.8**	420.1 ***	355.7 *** §	543.5 *** §	420.6*** §	185.4 ‡	178.2 ‡	357.9 *** §
	R2	264.1(60.6) ‡	350.2	449.9 *	368.3 +	470.0 **	571.5 ***	387.2* §	577.6 *** §	548.7 *** §
	R6	568.0(51.9) ‡	624.6	698.2 *	619.2	720.7 **	671.2 *	642.1	775.2 ***	743.7 **
**(d)**										
**Location**	**Component and Stages**	**Control**	**T1R1**	**T1R2**	**T2R1**	**T2R2**	**T1R1 & T2R1**	**T3R1**	**T3R2**	**T4R1**
**Fe/Zn** **(Zn** **Values)**	**Leaf**									
	V6	12.0(0.9) ‡	10.0 + ‡	10.9 ‡	12.2 ‡	15.1** ‡	13.7 ‡	11.8 ‡	11.5 ‡	11.9 ‡
	V14	40.6(25.0) ‡	138.0** §	93.4 §	96.0 + ‡	72.5 ‡	124.9* §	55.6 ‡	44.9 ‡	49.1 ‡
	V17	75.3(14.0) ‡	145.3***	149.9***	123.2* §	189.3*** §	186.0*** §	53.2 ‡	66.5 ‡	55.3 ‡
	R2	73.9(17.0) ‡	118.4 +	184.4***	103.6	158.8***	149.7**	82.7 §	139.3** §	64.8 ‡
	R6	64.3(6.8) ‡	66.8	104.9***	66.7	95.1**	100.9***	63.2	77.0	62.7 §
	**Stalk**									
	V6	13.7(1.8) ‡	8.8 + ‡	11.0 ‡	10.7 ‡	18.4 + ‡	11.9 ‡	8.6* ‡	11.0 ‡	11.9 ‡
	V14	67.2(8.5) ‡	90.4 + §	87.2 + §	97.5* ‡	66.7 ‡	93.4* §	64.9 ‡	58.5 ‡	71.4 ‡
	V17	119.0(14.7) ‡	116.3	154.8 +	124.2 §	140.01 §	132.3 §	102.6 ‡	103.6 ‡	86.7 + ‡
	R2	102.0(13.5) ‡	85.7	107.9	91.9	121.0	115.0	109.2 §	107.7 §	79.1 ‡
	R6	94.7(14.6) ‡	65.6	73.5	70.4	48.6*	74.8	77.9	45.5*	59.4 + §
	**Reproductive**									
	R2	65.8(5.7) ‡	64.9	66.0	74.9	70.0	72.1	74.3 §	72.0 §	72.3 ‡
	R6	54.4(6.7) ‡	56.4	63.8	56.6	65.7	77.3*	58.1	69.7 +	53.9 §
	**Grain**									
	R2	20.0(2.0) ‡	18.7	19.3	17.3	18.9	22.5	30.0 ** §	20.0 §	14.7 + ‡
	R6	210.0(14.3) ‡	206.9	213.4	194.7	212.3	241.6 +	209.6	220.4	206.5 §
	**Total**									
	V6	25.8(2.4) ‡	18.8* ‡	22.7 ‡	22.9 ‡	33.6 * ‡	24.9 ‡	20.4 ‡	22.5 ‡	23.8 ‡
	V14	107.7(28.5) ‡	228.5** §	180.6 + §	193.5* ‡	139.2 ‡	218.3 ** §	120.5 ‡	103.4 ‡	120.5 ‡
	V17	194.3(23.6) ‡	261.5*	304.8**	247.4 §	329.4 *** §	318.4*** §	155.8 ‡	170.1 ‡	141.9 ‡
	R2	261.7(25.0) ‡	287.8	377.6**	287.7	372.5 **	359.3 **	307.4 §	339.2* §	230.8 ‡
	R6	423.5(31.2) ‡	395.7	455.5	388.4	421.7	494.6	408.7	416.1	382.5 §
**(e)**										
**Location**	**Component and Stages**	**Control**	**T1R1**	**T1R2**	**T2R1**	**T2R2**	**T1R1 & T2R1**	**T3R1**	**T3R2**	**T4R1**
**Fe/Zn** **(Fe Values)**	**Leaf**									
	V6	144.9(12.1) ‡	110.2* ‡	121.6 ‡	124.4 ‡	170.4 ‡	145.8 ‡	130.1 ‡	136.8 ‡	130.8 ‡
	V14	247.7(32.9) ‡	311.8 §	248.0 §	294.3 ‡	276.4 ‡	315.0 ‡ §	235.4 ‡	259.5 ‡	281.4 ‡
	V17	351.0(32.1) ‡	380.6	374.3	339.7 §	434.7 + §	426.9 + §	266.3 + ‡	289.7 ‡	293.8 ‡
	R2	724.9(123.3) ‡	589.3	686.2	528.7	763.9	542.7	391.9** §	547.3 §	575.7 ‡
	R6	213.0(18.2) ‡	196.0	224.9	196.5	227.6	227.0	171.3 +	207.6	175.8 §
	**Stalk**									
	V6	47.2(7.0) ‡	37.7 ‡	35.5 ‡	35.3 ‡	46.8 ‡	44.6 ‡	29.8 + ‡	41.8 ‡	44.7 ‡
	V14	64.8(12.6) ‡	61.6 §	65.2 §	69.7 ‡	86.0 ‡	85.9 §	66.5 ‡	87.8 ‡	71.6 ‡
	V17	167.8(51.5) ‡	141.8	131.0	129 §	228.5 §	152.1 §	154.7 ‡	287.7 + ‡	155.6 ‡
	R2	436.0(63.1) ‡	230.5*	251.6*	208.1 *	372.4	341.7	330.2 §	260.6 + §	269.7 + ‡
	R6	116.1(24.8) ‡	125.0	130.8	108.0	114.4	164.8	171.9 +	169.2 +	170.9 + §
	**Reproductive**									
	R2	160.5(33.0) ‡	222.5	163.5	126.1	128.8	147.5	143.1 §	103.9 §	124.7 ‡
	R6	66.2(17.1) ‡	60.4	54.8	62.1	70.1	101.6	62.3	76.8	48.6 §
	**Grain**									
	R2	14.2(1.9) ‡	12.4	13.6	11.6	15.7	15.6	18.4 §	12.9 §	10.0 ‡
	R6	203.7(15.2) ‡	196.2	235.4	193.7	208.4	221.7	205.7	212.5	190.4 + §
	**Total**									
	V6	192.1(15.0) ‡	147.9* ‡	157.2 ‡	159.7 ‡	217.1 ‡	189.5 ‡	159.9 ‡	178.6 ‡	175.5 ‡
	V14	312.5(35.8) ‡	373.4 §	313.2 §	364 ‡	362.4 ‡	400.9 + §	301.9 ‡	347.3 ‡	353.0 ‡
	V17	518.8(69.0) ‡	522.4	505.3	468.8 §	663.2 §	579.0 §	421.0 ‡	577.4 ‡	449.3 ‡
	R2	1335.6(38.2) ‡	1054.7	1114.9	874.5*	1190.4	1047.5	923.5 + §	924.7* §	980.1 + ‡
	R6	599.1(32.7) ‡	577.5	645.8	560.6	620.5	715.2 *	611.2	630.5	585.7 §

† Least square mean plant nutrient quantity (g ha-1) followed by (SE for all values in the same row) and significant F test: Not Significant >0.10; + >0.05; * > 0.01; ** > 0.001; *** < 0.001. ‡ No foliar treatment had been applied at this stage. § First sampling following foliar treatment. ¶ First sampling following foliar treatment for plots with significant increase on grain yield. # First sampling following foliar treatment for plots with significant decrease on grain yield.

**Table 6:** LSMeans for Apparent Nutrient Recovery (ANR) at the end of the growing season (R6).

Location					
Treatment	Boron	Manganese	Zinc Only	Fe/Zn(Zn Values)	Fe/Zn(Fe Values)
ANR					
**T1R1 †**	5.8(2.9) ‡	7.1(3.7)	13.5(9.6)	−6.6(6.1)	−15.5(20.5)
**T1R2**	−0.3	11.9	15.5	3.8	5.4
**T2R1**	8.6	4.6	12.2	−8.3	−15.0
**T2R2**	−0.5	6.4	18.2	−0.2	6.6
**T1R1 and T2R1**	1.3	12.9	12.3	8.5	6.4
**T3R1**	0.8	9.9	17.6	−3.5	−38.0
**T3R2**	4.3	12.7	24.7	−0.5	−2.5
**T2R1 and T3R1**	0.3	10.4	20.9	-	-
**T4R1**	-	-	-	−9.8	−33.8
**T4R2**	-	-	-	0.2	−6.4
**T1R1 and T4R1**	-	-	-	−0.2	−12.7
**T3R1 and T4R1**	-	-	-	2.1	4.9

† T = Time of foliar application (1: early (V6–11), 2: middle (V15–18), 3: 4: late (R1–4)), R = Treatment rate (rate 1: lower level of industry recommendation & rate 2: upper level of industry recommendation). ‡ ANR Least Square Mean followed by (SE for all values in the same column).

**Table 7:** Multiple comparison test of LSmean R6 Biomass (Mg ha^−1^) comparing treatment effects with control using Dunnett’s Test.

	Foliar Micronutrient Treatment Locations			
Treatments	Boron	Manganese	Zinc Only	Fe & Zn
**Control**	31.70(0.99) †	19.38(1.11)	31.35(1.19)	19.47(0.63)
**T1R1 ‡**	32.16	22.74 **	29.69	20.86
**T1R2**	32.14	20.98	30.61	19.89
**T2R1**	31.43	21.54 +	31.79	20.51
**T2R2**	32.76	21.80 *	30.58	20.12
**T1R1 and T2R1**	31.01	20.41	31.48	19.83
**T3R1**	31.86	19.92	29.68	20.13
**T3R2**	30.90	19.81	30.01	20.04
**T2R1 and T3R1**	31.75	20.65	30.43	-
**T4R1**	-	-	-	19.16
**T4R2**	-	-	-	19.89
**T1R1 and T4R1**	-	-	-	20.92
**T3R1 and T4R1**	-	-	-	20.25

† LSmean R6 biomass (Mg ha^−1^) followed by (SE for all values in the same column) and significant F test: Not Significant >0.10; + >0.05; * >0.01; ** >0.001. ‡ T = Time of foliar application (1: early (V6–V11), 2: middle (V15–V18), 3: 4: late (R1–R4)), R = Treatment rate (rate 1: lower level of industry recommendation & rate 2: upper level.

## Supplementary Materials

The following are available online at https://www.mdpi.com/2223-7747/10/3/528/s1, Table S1: Schedule of whole plant sampling used for uptake, partitioning, and translocation analysis at different locations, Table S2: Schedule of foliar treatment†s applied at various rates and times at different locations., Table S3: Summary location characteristics and results to each foliar micronutrient treatment, Figure S1: Maize growth stages (Ciampitti et al. [46]).

## Abbreviations

V (1–18)	Vegetative growth stage
VT	Tasseling stage
R (1–6)	Reproductive growth stage
RCBD	Randomized Complete Block Design
ANOVA	Analysis of Variance
ANR	Apparent Nutrient Recovery
T	Time of foliar application (1: early (V6–11), 2: middle (V15–18), 3: 4: late (R1–4))
R	Treatment rate (rate 1: lower level of industry recommendation & rate 2: upper level of industry recommendation)
*p*-value	Probability
