# Supplementary material for: Effect of Foliar Micronutrients (B, Mn, Fe, Zn) on Maize Grain Yield, Micronutrient Recovery, Uptake, and Partitioning
Source: Plants (Basel). 2021 Mar 11;10(3):528. doi: 10.3390/plants10030528 (PMC8001809; doi:10.3390/plants10030528)
Supplement: Supplementary file 1 [file plants-10-00528-s001.pdf]

Effect of Foliar Micronutrients (B, Mn, Fe, Zn) on Maize Grain Yield, Micronutrient Recovery, Uptake, and Partitioning

Zachary P. Stewart [1,2,3,4,*], Ellen T. Paparozzi [2], Charles S. Wortmann [2], Prakash Kumar Jha [3] and Charles A. Shapiro [2,5]

Table S1. Schedule of whole plant sampling used for uptake, partitioning, and translocation analysis at different locations.

	2014				2015
Sampling Date	Location 1 (Foliar B#)	Location 2 (Foliar Mn‖)	Location 3 (Foliar Zn**)	Location 4 (Foliar Fe & Zn††)	Location 5 (Foliar Fe‡‡)
	Date (Growth Stage)				
Planting Date	April 25	May 7	May 13	May 13	April 25
Diagnostic Soil and Leaf Sample‡	June 18 (V5)	June 18 (V5)	June 18 (V5)	June 18 (V5)	June 26 (V6)
Plant Sampling 1§	June 23 (V6)	June 21 (V7)	June 21 (V6)	June 21 (V6)	-
Plant Sampling 2§	July 10 (V13)	July 11 (V15)	July 15 (V14)	July 14 (V14)	-
Plant Sampling 3§	July 21 (V17)	July 20 (VT)	July 25 (V17)	July 24 (V17)	-
Plant Sampling 4§	August 5 (R2)	August 4 (R3)	August 7 (R2)	August 6 (R2)	-
Plant Sampling 5§	October 20 (R6)	October 6 (R6)	October 9 (R6)	October 10 (R6)	-
Harvest Date	November 8 (R6)	November 7 (R6)	November 18 (R6)	November 9 (R6)	November 25 (R6)

† All foliar treatments were applied to 9.1m x 3.05m plots (4 rows with 76.2cm spacing) with a backpack sprayer with four nozzles at a height of 0.3m above the canopy with a band width of 0.38m

‡ Initial soil and leaf samples were collected to determine if treatments were needed and which nutrient may be needed

§ Whole plant samples were collected and partitioned into leaf, stalk, reproductive, and grain where applicable.

Table S2. Schedule of foliar treatments† applied at various rates and times at different locations.

¶ T = Time of foliar application (1 : early (V6-11), 2: middle (V15-18), 3: 4: late (R1-4)), R = Treatment rate (rate 1: lower level of industry recommendation & rate 2: upper level of industry recommendation)
Boron rate 1: 1,000 mg kg^{-1} or 0.14 kg B ha^{-1} rate 2: 2,000 mg kg^{-1} or 0.28 kg B ha^{-1}; Manganese rate 1: 5,207 mg kg^{-1} or 0.73 kg Mn ha^{-1} rate 2: 10,413 mg kg^{-1} or 1.46 kg Mn ha^{-1};
Zinc rate 1: 3,000 mg kg^{-1} or 0.42 kg Zn ha^{-1} rate 2: 6,000 mg kg^{-1} or 0.84 kg Zn ha^{-1}; Iron rate 1: 750 mg kg^{-1} 0.11 kg Fe ha^{-1} rate 2: 1500 mg kg^{-1} 0.22 kg Fe ha^{-1}
8.0% Boron derived from boric acid and contains proprietary surfactants, saccharides, and antifoaming solvents CornSorb: MAX-IN® Boron (WinField Solutions: St. Paul, MN)

Foliar Treatment (Time and Rate)	2014				2015
	Location 1 (Foliar B#)	Location 2 (Foliar Mn∣∣)	Location 3 (Foliar Zn**)	Location 4 (Foliar Fe & Zn††)	Location 5 (Foliar Fe‡‡)
	Date (Growth Stage)				
T1R1¶	July 2 (V10)	July 2 (V11)	July 8 (V11)	July 8 (V10)	June 26 (V6)
T1R2¶	July 2 (V10)	July 2 (V11)	July 8 (V11)	July 8 (V10)	June 26 (V6)
T2R1¶	July 13 (V15)	July 13 (V18)	July 16 (V15)	July 16 (V15)	July 17 (V15)
T2R2¶	July 13 (V15)	July 13 (V18)	July 16 (V15)	July 16 (V15)	July 17 (V15)
T1R1 and T2R1‡	July 2 (V10) & July 13 (V15)	July 2 (V11) & July 13 (V18)	July 8 (V11) & July 16 (V15)	July 8 (V10) & July 16 (V15)	June 26 (V6) & July 17 (V15)
T3R1¶	July 28 (R1)	July 26 (R2)	July 28 (R1)	July 28 (R1)	August 8 (R2)
T3R2¶	July 28 (R1)	July 26 (R2)	July 28 (R1)	July 28 (R1)	August 8 (R2)
T2R1 and T3R1¶	July 13 (V15) & July 28 (R1)	July 13 (V18) & July 26 (R2)	July 16 (V15) & July 28 (R1)	-	July 17 (V15) & August 8 (R2)
T4R1¶	-	-	-	August 19 (R4)	-
T4R2¶	-	-	-	August 19 (R4)	-
T1R1 and T4R1¶	-	-	-	July 8 (V10) & August 19 (R4)	-
T3R1 and T4R1¶	-	-	-	July 28 (R1) & August 19 (R4)	-

∣∣ 15.62% Manganese sulfate in addition to proprietary surfactants, saccharides, and antifoaming solvents CornSorb: MAX-IN® Ultra Manganese (WinField Solutions: St. Paul, MN)
** 9.0% ZnEDTA (zinc-ethylenediaminetriacetate) and contains proprietary surfactants, saccharides, and antifoaming solvents CornSorb: Origin® Zinc 9% (WinField Solutions: St. Paul, MN)
†† Contains both 4.5% FeHEDTA and 9.0% ZnEDTA
‡‡ 4.5% FeHEDTA (iron-hydroxyethylenediaminetriacetate) in addition to proprietary surfactants, saccharides, and antifoaming solvents CornSorb: ULTRA-CHE IRON 4.5% HEDTA (WinField Solutions: St. Paul, MN)

Table S3. Summary location characteristics and results to each foliar micronutrient treatment.

Experimental Focus.	**Low-Deficient Leaf Tissue**	**High-Excessive Leaf Tissue**	**Low-Deficient Soil**	**Change in applied nutrient concentration due to treatment**	**Change in applied nutrient uptake due to treatment**	**Yield Response**	**Target Growth Stage‡**
**B**	+ +†	-	- -	+ +	+ +	0	Pre-V10
**Mn**	+	-	- - -	+ +	+ +	+	Vegetative
**Zn only**	0	+	- - -	+ +	+ +	-	Any
**Fe / Zn**	- - / +	+ + +/ -	- - - / - - -	- - / + +	- - / + +	0	Undefined
**Fe only**	+ + +	-	- -	no data	no data	+ + +	Any

† +/0/- indicates direction of agreement with statement and number of characters indicates magnitude. (i.e. + indicates agreement and - indicates disagreement whereas 0 indicates neutral)
‡ Under conditions of confirmed deficiency of the target micronutrient, the listed growth stage would be the recommended stage for foliar application of the specified micronutrient.

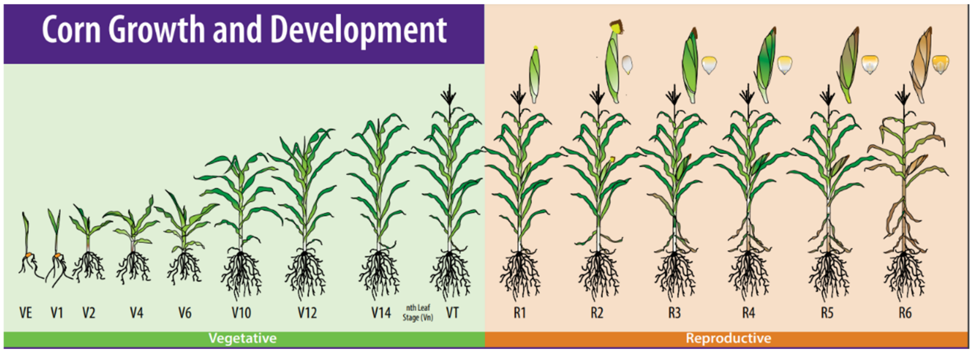

Figure S1. Maize growth stages (Ciampitti et al. [46]).
